# The Effectiveness of a Multidisciplinary Integrative Survivorship Program for Cancer-Related Cognitive Impairment: A Prospective Cohort Study

**DOI:** 10.3390/cancers18050785

**Published:** 2026-02-28

**Authors:** Nathalie Vanlaer, Camille Testaferrata, Lynn Decabooter, Iris Dirven, Cleo Bertels, Lara Stas, Sebastien Van Eycken, Matthieu Hein, Bart Neyns, Anne Rogiers

**Affiliations:** 1 Department of Medical Oncology, Vrije Universiteit Brussel (VUB), Translational Oncological Research Centre, Universitair Ziekenhuis Brussel, 1090 Brussels, Belgiumanne.rogiers@chu-brugmann.be (A.R.); 2 Core Facility, Support for Quantitative and Qualitative Research (SQUARE), Vrije Universiteit Brussel (VUB), 1090 Brussels, Belgium; 3 Department of Psychiatry, Centre Hospitalier Universitaire Brugmann, 1020 Brussels, Belgium; 4 Laboratory of Medical Psychology and Addictology (ULB312), Université Libre de Bruxelles, 1020 Brussels, Belgium

**Keywords:** cancer-related cognitive impairment, cancer survivors, cognition, survivorship, psycho-oncology, multidisciplinary, integrative, intervention, cognitive complaints, psychological distress

## Abstract

**Background**: Cancer-related cognitive impairment (CRCI) affects quality of life, daily functioning and return-to-work. However, CRCI remains under-addressed in cancer care. Since cognitive complaints often co-occur with fatigue and psychological distress, a multimodal approach is warranted. We developed Integrative Neuro-Cognitive Remediation Therapy (INCRT), a multidisciplinary survivorship program combining personalized cognitive function and strategy training with group-based psychoeducation, cognitive–behavioral therapy and Acceptance and Commitment Therapy, and onco-yoga. **Methods**: Cancer survivors suffering from CRCI were eligible. Assessments included neuropsychological testing, patient-reported outcomes, and daily functioning at baseline (T0), post-intervention (T1), and 6-month follow-up (T2). Primary outcomes were objective and subjective neurocognitive functioning (NCF); secondary outcomes were psychological distress, fatigue, metacognition, and daily functioning. Changes were analyzed using linear mixed models. **Results**: Between November 2022 and January 2025, 44 of 56 eligible survivors enrolled; 38 completed the program (71.1% female; median age 53.5). Objective and subjective NCF improved significantly at T1 and T2 (*p*s < 0.001). Psychological distress, fatigue, and unhelpful metacognitions decreased over time (*p*s < 0.05). Participants reported greater emotional and cognitive insight and improved daily functioning. **Conclusions**: INCRT improves cognitive functioning, reduces psychological distress and fatigue, and enhances daily functioning, with benefits maintained at follow-up. The integrative design supports sustained effects by promoting internalization and daily application of learned strategies.

## Simple Summary

Many cancer survivors experience difficulties with memory, attention, and mental processing, referred to as cancer-related cognitive impairment (CRCI). These challenges can impact daily functioning and returning to work, and frequently occur alongside fatigue and psychological distress. To address these interconnected problems, we developed Integrative Neuro-Cognitive Remediation Therapy (INCRT), a multimodal survivorship program combining individualized cognitive function and strategy training with psychoeducation, psychological therapies, and onco-yoga. We report on the first 38 survivors who completed the program. Patients showed significant improvements in both objectively measured and self-reported cognitive functioning immediately after the program and six months later. They also reported reductions in psychological distress, fatigue, and unhelpful thinking patterns, as well as improved insight into their emotions and daily functioning. These findings suggest that INCRT is a promising approach for CRCI and overall well-being, and its integrative design supports maintained effects through the implementation of the learned strategies in daily life.

## 1. Introduction

Advances in cancer diagnostics and treatment have significantly improved survival rates, resulting in a growing population of cancer survivors [1,2,3]. However, a considerable proportion of cancer survivors experience persistent neurocognitive and psychosocial challenges that diminish health-related quality of life (HRQoL), have an impact on return-to-work and daily functioning [4,5,6,7,8]. Among these late treatment effects, cancer-related cognitive impairment (CRCI) has emerged as an important aspect of survivorship care [9].

CRCI encompasses subjective cognitive complaints and objective neurocognitive impairment (NCI), respectively assessed by patient-reported outcome measures (PROMs), and measured by neuropsychological tests. In non-central nervous system (non-CNS) cancers, deficits most often involve processing speed, attention, memory, and executive functioning [9,10,11]. The pooled prevalence of objective NCI ranges from 21 to 34% [12,13], whereas 44–75% of cancer survivors report subjective complaints across cancer types and treatments [7,12,14].

Beyond CRCI, cancer survivors frequently experience a wide range of symptoms including fatigue, pain and psychosocial issues. The latter includes anxiety, depression, fear of recurrence, and diminished social functioning [8,15,16,17,18]. Subjective cognitive complaints are strongly correlated with these symptoms, whereas their correlation with objective test performance is generally weak [14,19,20,21]. This underscores the need to address both neurocognitive and psychosocial factors in an integrative, multidisciplinary survivorship care program to tackle CRCI.

Multiple non-pharmacological approaches have been investigated to address CRCI, including physical exercise; computerized cognitive training, compensatory strategy training, or their combination; psychological and mind–body interventions; and multimodal programs integrating several of these components [19,22].

A recent umbrella review including 64 systematic reviews found that cognitive training and cognitive rehabilitation showed the strongest and most consistent benefits, with small-to-moderate improvements in both subjective and objective cognition. The review concluded that multimodal interventions showed promise although treatment modalities varied considerably [22]. Programs using solely computerized “brain training” in non-CNS survivors have reported mixed results [19]. It is argued that cognitive training alone warrants limited transfer to everyday functioning, whereas coupling training with personalized, real-life goals appears to enhance functional gains [23,24].

Evidence from psychiatric research further supports the value of combining cognitive training with compensatory strategies. Cognitive Remediation Therapy (CRT), a behavioral intervention targeting attention, memory, executive functions, metacognition, and social cognition [25], has shown durable effects on global cognition and functioning. Meta-analyses indicate larger effects when cognitive training is integrated with strategy training compared to cognitive training alone [25,26].

Within cancer survivorship care, aerobic and resistance training have demonstrated benefits for subjective and objective cognitive outcomes [19] and for global QoL [27]. These benefits are thought to arise from brain-derived neurotrophic factor-mediated neurogenesis and plasticity, improvements in cardiovascular fitness and insulin regulation, and reductions in inflammation [19,28,29].

Psychoeducational and psychological approaches also contribute meaningfully to CRCI management. Psychoeducation can reduce fear of cancer recurrence, stress, and self-reported cognitive problems [30] and cognitive complaints [31]. Cognitive behavioral therapy (CBT) has demonstrated efficacy in reducing fear of cancer recurrence, anxiety, depression, and HRQoL [27,32,33]; while Acceptance and Commitment Therapy (ACT) reduces anxiety, depression, and fear of recurrence and improves psychological flexibility and QoL [34]. Mindfulness-based interventions have shown efficacy in reducing emotional distress, subjective cognitive complaints, fatigue, and HRQoL [35,36]. Yoga has also shown improvements in global QOL and fatigue [27,37].

Despite these promising findings within cancer survivorship research, most interventions target isolated symptoms or patient subgroups. Research on multimodal programs in clinical settings that address both neurocognitive and psychosocial challenges across diverse cancer populations remains limited [9]. We developed Integrative Neuro-Cognitive Remediation Therapy (INCRT), a multimodal intervention that combines psychoeducation (including lifestyle guidance on sleep, diet, and physical activity), CBT, ACT, personalized computerized cognitive training, CRT-based strategy training, and onco-yoga. The aim is to target neurocognitive and psychosocial outcomes and daily functioning.

This study evaluates the efficacy of INCRT in improving (1) objective neurocognitive functioning (NCF) and (2) subjective NCF in cancer survivors with diverse diagnoses and treatment histories. Secondary outcomes include psychological distress, fatigue, metacognitive beliefs, and daily functioning. We hypothesized that the multidisciplinary and integrative nature of INCRT would lead to clinically meaningful improvements in both primary and secondary endpoints. We expect this effect to arise from a synergistic process among psychological state, cognitive functioning, and fatigue, in which improvements in psychological state and cognitive functioning, together with reductions in fatigue, are interconnected and mutually reinforcing.

## 2. Materials and Methods

### 2.1. Study Participants and Procedures

Eligible participants were cancer survivors who (a) had objective NCI according to the International Cancer and Cognition Task Force (ICCTF) criteria [38] and/or subjective cognitive complaints (self-reported complaints with impact on daily life); (b) were mentally and physically able to participate in group sessions; (c) were disease-free after non-CNS tumors or had no active disease for CNS tumors; (d) had received any type of cancer therapy; (e) had completed treatment (immunotherapy, chemotherapy, radiotherapy, surgery, etc.) except for maintenance treatments such as anti-hormonal therapy; and (f) were Dutch- or French-speaking. Exclusion criteria included severe non-cancer-related psychiatric disorders and cognitive impairment related to dementia.

This study was approved by the central institutional Committee of Medical Ethics of the Universitair Ziekenhuis Brussel (UZ Brussel) and the local Committee of Medical Ethics of the Centre Hospitalier Universitaire Brugmann (CHU Brugmann) in 2022 (CME: 2022-043). All patients provided their written informed consent prior to study enrollment. The assessments were conducted at UZ Brussel, and the INCRT program was delivered at CHU Brugmann in a day hospital setting.

This prospective exploratory cohort study comprises three assessments: baseline before the start of INCRT (T0), post-INCRT (T1), and 6-month follow-up (T2) (ClinicalTrials.gov: NCT05667857). All assessments consisted of neuropsychological testing, patient-reported outcome measures (PROMs), and an assessment of daily functioning. Sociodemographic information was gathered through a general questionnaire. Information on medical history was retrieved from hospital patient files. Recruitment was based either on referrals from hospital-affiliated and external healthcare providers, or through information and flyers distributed to patient organizations, where patients could directly contact the study coordinator.

#### Integrative Neuro-Cognitive Remediation Therapy (INCRT)

The INCRT program is a multimodal intervention delivered once weekly in a day hospital setting. The multimodal nature of the intervention was specifically designed to address multiple interacting dimensions of cancer-related cognitive impairment (e.g., cognitive, psychological, and physical components). It was initially implemented as a 12-week program and subsequently as an 8-week program (one full day per week from 9:00 to 16:30), with identical content across both formats. The program was delivered by a multidisciplinary team consisting of a psychiatrist, neuropsychologist, psychologist, social nurse, survivorship care plan coordinator, and certified yoga instructor. Each therapy day consisted of four core components: (1) psychoeducation (e.g., lifestyle guidance), combined with CBT and ACT elements (e.g., addressing procrastination, self-esteem, and cognitive distortions) (3 h per therapy day); (2) personalized computerized cognitive training (40 min per therapy day); (3) Cognitive Remediation Therapy (CRT)–based strategy training (40 min per therapy day); and (4) onco-yoga (Hatha yoga adapted to patients’ physical abilities, incorporating relaxation) (1 h per therapy day). At the end of each therapy day, patients were assigned homework exercises to facilitate transfer of the learned strategies to daily life (e.g., applying strategies to everyday tasks).

### 2.2. Neuropsychological Testing

Objective NCF was measured using a computerized neurocognitive test battery (COGBAT^®^ and CORSI^®^, Vienna Test System), and pencil-and-paper neuropsychological tests. The selected tests measure processing speed, attention, memory, and executive functioning, as recommended by the ICCTF guidelines [38]. Pencil-and-paper neuropsychological tests comprises California Verbal Learning Test (CVLT-II) (verbal learning and Long-Term Memory (LTM)), WAIS-IV symbol search and coding (processing speed), and WAIS-IV Digit Span forward and backward (verbal short-term memory span and working memory). The COGBAT^®^ test battery consists of Trail Making Test—A and B (processing speed and cognitive flexibility), Perception and attention functions test (alertness and divided attention), Figural Memory Test (FMT) (visual learning and LTM, Tower of London Test (planning capacity), N-Back Test (working memory), Response Inhibition, and Corsi Block Tapping Test (visuo-spatial short-term memory span). An internal consistency of Cronbach α = 0.63–0.91 has been reported previously [39]. Alternate forms for the COGBAT^®^ and the CVLT-II were used to minimize practice effects. Neurocognitive test scores were transformed into age-adjusted z-scores. We classified NCI according to the ICCTF guidelines, i.e., ≤−1.5 sd on at least 2 subtests, or ≤−2.0 sd on at least 1 subtest [38].

### 2.3. Patient-Reported Outcome Measures (PROMs)

*Subjective cognitive complaints.* The Cognitive Failures Questionnaire (CFQ) is a validated PROM consisting of 25 items, ranging from 0 to 100, and measures lapses in cognition in everyday tasks [40]. We use the cutoff point of ≥44 to identify moderate cognitive complaints [41]. The CFQ showed excellent internal consistency (α = 0.906; 95% CI = 0.856–0.944) in our sample.

*Emotional distress.* The Hospital Anxiety and Depression Scale (HADS) is a validated instrument to measure the severity of anxiety and depression symptomatology in a hospital setting. It consists of 14 items, with a HADS-anxiety and HADS-depression subscale [42]. On both subscales, a cutoff of ≥8 was used [43]. The HADS-total score was used to measure longitudinal changes in emotional distress and showed good internal consistency (α = 0.843; 95% CI = 0.759–0.908) in our sample.

*Fatigue.* The Fatigue Severity Scale (FSS) is a validated instrument to measure fatigue, consisting of 9 items on a 7-point Likert scale. A mean score of ≥4 indicates clinical levels of fatigue [44]. The FSS showed excellent internal consistency (α = 0.934; 95% CI = 0.898–0.962) in our sample.

*Metacognitive beliefs.* The Metacognitions Questionnaire 30 (MCQ-30) is a 30-item questionnaire on a 4-point Likert scale. It consists of 5 subscales and a total score. The total score has a range of 30 to 120, in which a higher score indicates higher levels of unhelpful maladaptive cognitions. It has good test–retest reliability and has been validated in the oncological population [45,46]. The total score showed an excellent internal consistency (α = 0.910; 95% CI = 0.864–0.947) in our sample.

#### Exploratory Variables

*Anxiety-state.* The State-Trait-Anxiety Inventory (STAI-Y-1) is a validated measure for state anxiety, consisting of 20 four-point scale items. Higher scores align with more anxiety symptoms [47]. It has an excellent internal consistency (α = 0.952; 95% CI = 0.863–0.996) in our sample.

*Fear of cancer recurrence.* The Fear of Cancer Recurrence Inventory Short Form (FCRI-SF) has 9 items, ranging from 0 to 36, with higher scores indicating more FCR severity [48]. We used the cutoff of ≥13 to identify clinical FCR and also report two other cutoff points: ≥16, which has higher specificity and ≥22, which evaluates pathological FCR [15,49]. It has a good internal consistency (α = 0.881; 95% CI = 0.815–0.931) in our sample.

*Depressive symptoms.* The Beck Depression Inventory (BDI-II) is a self-report questionnaire consisting of 21 questions. It measures the symptoms for depression according to the criteria of the DSM-IV [50]. A previous study reported at a retest reliability ranging from 0.73 to 0.96 and good criterion validity [51]. It has a good internal consistency (α = 0.805; 95% CI = 0.703–0.885) in our sample.

*Procrastination.* The Pure Procrastination Scale (PPS) is a validated instrument, consisting of 12 five-point scale items, with higher scores indicating a higher tendency to procrastinate [50]. It has an excellent internal consistency (α = 0.904; 95% CI = 0.851–0.943) in our sample.

*Perfectionism.* The Frost Multidimensional Perfectionism Scale (FMPS) is a validated instrument, consisting of 35 items, in which the total score indicates a higher tendency for perfectionism [52]. It has an excellent internal consistency (α = 0.947; 95% CI = 0.920–0.968) in our sample.

### 2.4. Daily Functioning Assessment

Various domains of daily functioning were assessed through a structured interview. The topics covered in the interview were doing household tasks, administration, social interactions, leisure, and professional activity, the perceived impact of the INCRT program on dietary changes, the understanding of their emotions and cognitions, and predefined personalized goals. The evaluation of personalized goals was done in parallel by two investigators, of whom one was not implied during the INCRT program and therefore had a neutral position in evaluating the results of the goals.

### 2.5. Statistical Analysis

All PROMs were verified for completeness during the evaluations. Statistical analysis was performed using SPSS running on version 29.0.0.0 (IBM, Armonk, NY, USA). Composite scores for objective neurocognitive functioning (NCF) were calculated by averaging the standardized neuropsychological test scores, with certain tests reversed so that higher scores consistently reflected better neurocognitive performance (Table S1). Cronbach’s alpha was calculated to check the reliability of the composite scores.

Descriptive statistics were expressed as the mean and SD, and the percentage of clinical levels of the PROMs at all timepoints using their validated cutoff scores. Changes on neuropsychological tests were assessed descriptively using the Reliable Change Index (RCI), in which a score of ≥1.96 indicates a clinically significant change [53]. Pearson correlation was performed between PROMs and objective NCF at baseline.

Linear mixed model (LMM) analyses were conducted to examine changes over time and between intervention groups. A separate LMM was estimated for each dependent variable: objective neurocognitive function (NCF), subjective cognitive complaints, psychological distress (HADS-total), fatigue (FSS), and metacognitive beliefs (MCQ-30). Each model included the fixed effects of time (baseline (T0), post-INCRT (T1), 6-month follow-up (T2)), group (8-week vs. 12-week INCRT), and their interaction, with an AR(1) covariance structure to account for within-subject correlations. For the model assessing objective NCF, age and education level were added as covariates.

## 3. Results

**Figure 1:**
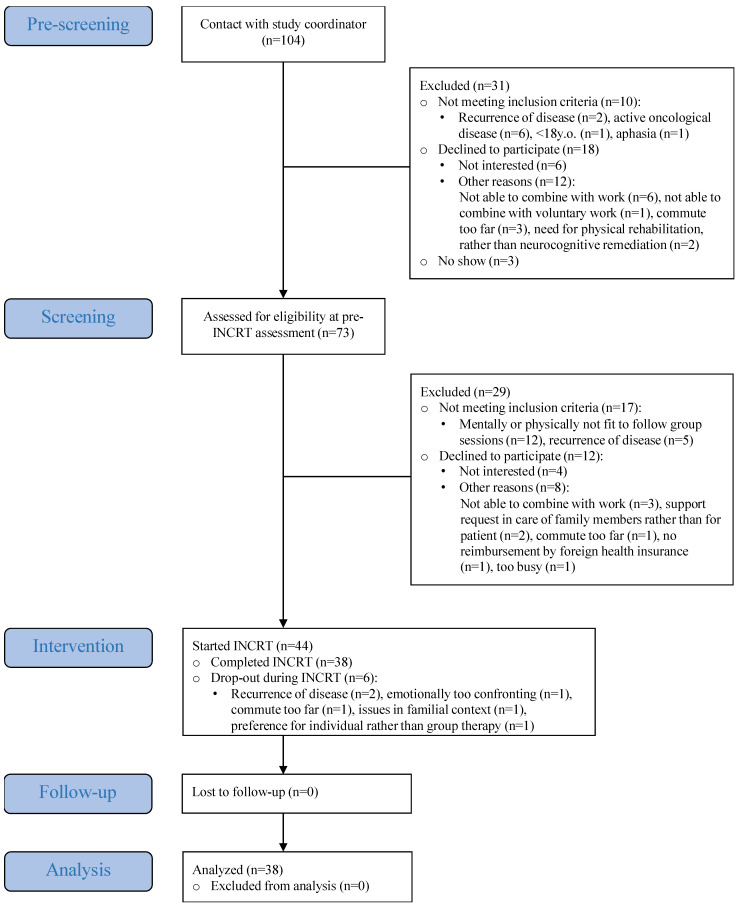
Consort diagram.

Following referral or self-referral, 104 patients underwent an initial brief pre-screening and a subsequent comprehensive screening. Of these, 27 did not meet the inclusion criteria, 30 declined participation, and 3 were no-shows, resulting in 44 patients initiating the INCRT intervention. Thirty-eight participants completed the program between November 2022 and January 2025 (12-week INCRT: *n* = 13; 8-week INCRT: *n* = 25) and were included in the analysis (Figure 1). Most participants were female (*n* = 27, 71%), with a median age of 53.5 years [30 y–77 y]. The most common cancer types were breast cancer (*n* = 15, 39%) and melanoma (*n* = 10, 26%) (Table 1). Attendance was high (mean = 95%, range = 75–100%), with most patients attending all sessions (*n* = 28, 74%).

### 3.1. Objective Neurocognitive Functioning

**Figure 2:**
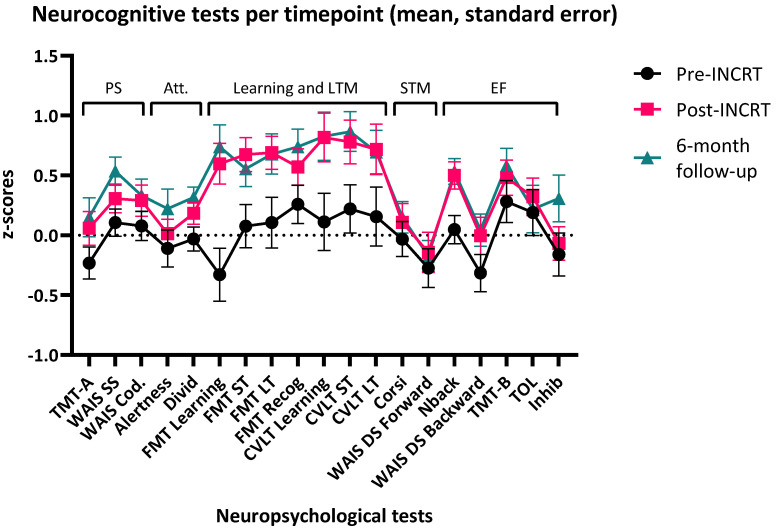
Line plot with mean and standard error of neurocognitive test results per timepoint for each neuropsychological test. Note. z-scores are calculated to have a mean of 0 and standard deviation of 1. PS = Processing Speed; Att. = Attention; LTM = Long-Term Memory; STM = Short-Term Memory; EF = Executive Functioning; WAIS SS = WAIS Symbol Search; WAIS Cod. = WAIS coding; Divid = Divided attention; ST = Short-Term Recall; LT = Long-Term Recall; Recog = Recognition; WAIS DS = WAIS Digit Span; Inhib = Inhibition.

Figure 2 and Table S2 provide the descriptive statistics for objective NCF.

There was a reduction in objective NCF impairment over time, with 14 patients (37%) demonstrating objective NCF impairment at T0 to 3 patients (8%) at T1 and 5 patients (13%) at T2. The RCI showed that most clinically significant improvements were seen in the FMT learning ability, and the WAIS-IV Digit Span forward and backward, with ≥10 patients (26.3%) having a clinically significant improvement on either or both timepoints (Table S3). The two glioma patients in our sample improved on at least one neuropsychological test at T1 and T2 according to the RCI (Table S4).

Results of the LMM showed that time was significantly associated with objective NCF (*p* < 0.001), with scores showing an improvement at both T1 and T2 as compared to T0 (both *p* < 0.001). There was no group or interaction effect between time and group (Table 2).

Exploratory analyses showed that longer time since remission was associated with significantly greater improvement in objective NCF from T0 to T1 (β = 0.033, *p* = 0.014), but not from T0 to T2 (β = 0.022, *p* = 0.223). Female participants showed significantly greater improvement than male participants from T0 to T1 (β = −0.230, *p* = 0.014). At T2, this gender difference was no longer statistically significant (β = −0.097, *p* = 0.443). Age and education level were not associated with differential improvement over time (Table S5).

### 3.2. Subjective Cognitive Complaints

Descriptive statistics showed that the number of patients with increased cognitive complaints reduced from 65.8% (*n* = 25) at T0 to 36.8% (*n* = 14) at T1, and 34.2% (*n* = 13) at T2.

The LMM showed that time was significantly associated with subjective NCF (*p* < 0.001), with scores showing a reduction in subjective cognitive complaints at both T1 and T2 as compared to T0 (both *p* < 0.001) (Table 2).

No significant interaction between time and group was found, indicating that the pattern of change over time did not differ significantly between the 8- and 12-week groups. A significant effect of group was present, reflecting a stable difference in subjective NCF across all timepoints between groups (Table 2).

Significant positive correlations between subjective NCF, all PROMs and objective NCF were found (*p*s < 0.05) (Table S6).

### 3.3. Daily Functioning and Return-to-Work

All patients achieved at least one of their personalized goals at both T1 and T2. The most frequently predefined goals were work resumption (*n* = 14) and functioning at work (*n* = 12), starting a hobby (*n* = 14) and sports activity (*n* = 12), and self-care (*n* = 12) (Table S7).

All patients reported a better understanding of their emotions and cognitions and improved in daily functioning, mostly in single tasking, reading, setting boundaries, following group conversations, household tasks, and coping with unexpected situations (Table 3). Some patients had a delayed improvement (improvement at T2, but not at T1), such as in household cleaning, reading, leisure activities, and following group conversations.

Out of 27 patients that were on full-time or part-time sick leave before INCRT, respectively 3 and 9 patients had increased their work capacity at T1 and T2. Respectively, 10 and 6 patients were in a return-to-work process but had not yet resumed work at T1 and T2. All patients who had set the goal to resume work, or start a return-to-work process, attained it at the 6-month follow-up (Table S7).

### 3.4. Psychological Functioning

**Figure 3:**
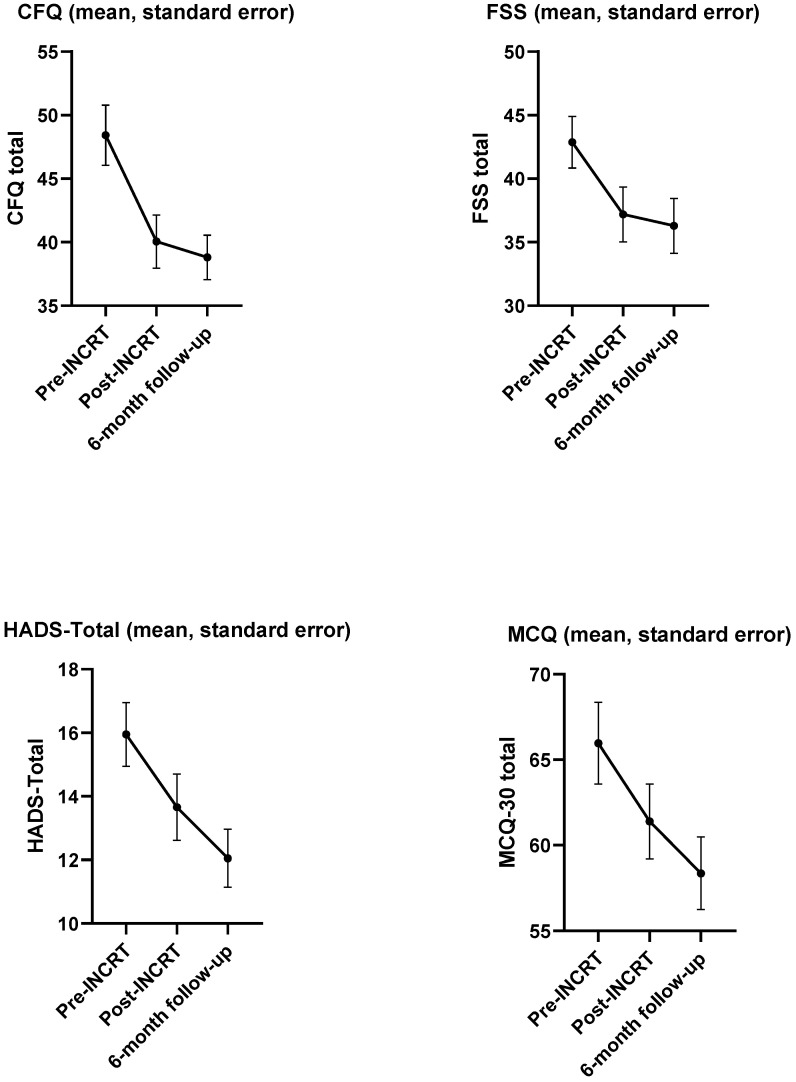
Line plot of means and standard error per PROM over time. CFQ = Cognitive Failures Questionnaire; HADS = Hospital Anxiety and Depression Scale; FSS = Fatigue Severity Scale: MCQ-30 = Metacognition Questionnaire-30.

Descriptive statistics showed that the number of patients with elevated levels of psychological distress, fatigue and unhelpful metacognitive beliefs decreased over time (psychological distress T0: *n* = 31 (81.6%); T1: *n* = 21 (55.3%), T2: *n* = 19 (50%); fatigue: T0: *n* = 30 (78.9%), T1: *n* = 22 (57.9%), T2: *n* = 20 (52.6%); unhelpful metacognitive beliefs: T0: *n* = 12 (31.6%), T1: *n* = 8 (21.1%), T2: *n* = 6 (15.8%)) (Figure 3 and Table 4).

The LMM demonstrated that time was significantly associated with emotional distress (Model 3: *p* = 0.004), fatigue (Model 4: *p* = 0.041), and metacognitive beliefs (Model 5: *p* < 0.001), with scores showing a reduction in emotional distress (Model 3: *p*s < 0.05) and fatigue (Model 4: *p*s < 0.001) at both T1 and T2 compared to T0, and a significant reduction in maladaptive metacognitions at T2 compared to T0 (Model 5: *p* = 0.01) (Table 2).

No significant interaction between time and group was found for emotional distress, indicating that the pattern of reduction over time did not differ significantly between the 8- and 12-week groups. A significant effect of group was present, reflecting a stable difference in emotional distress across all timepoints between groups (Model 3, Table 2).

Figure S1 visualizes the group differences over time for FSS (Model 4). It visualizes that at baseline, the 8-week INCRT group had higher FSS scores than the 12-week INCRT group. No significant differences between the groups were found at post-INCRT (T1) or at 6-month follow-up (T2). For maladaptive metacognitions (Model 5, Table 2), no group differences and interaction effects were found.

Regarding exploratory outcomes, there were significant associations between time and anxiety (*p* < 0.001) and depression symptoms (*p* < 0.001), perfectionism (*p* = 0.007), procrastination (*p* < 0.001), and the cognitive confidence subscale of the MCQ-30 (*p* < 0.001), with scores showing an improvement at both T1 and T2 as compared to T0, but not between time and fear of cancer recurrence (Table S8 and Figure S2).

## 4. Discussion

Our multidisciplinary integrative survivorship program resulted in significant and maintained improvements in cognitive, psychological and daily functioning. Importantly, our intervention found improvements in both subjective cognitive complaints and objective neuropsychological test results assessed immediately after the program; moreover, patients continued to improve at 6-month follow-up. There were no meaningful differences between the 8-week and 12-week program, suggesting a preference towards the 8-week program given its higher acceptance rate of the participants.

These promising results can be explained by the integration of several approaches that have each demonstrated individual efficacy in improving either cognitive or psychological outcomes [25,27,33,34,37]. Previous studies have demonstrated beneficial effects of isolated interventions, including computerized cognitive training, compensatory strategy training, physical exercise, CBT, ACT, mindfulness-based interventions, and yoga [25,27,33,34,37]. Among these, computerized cognitive training and compensatory strategy training, have shown the most consistent improvements in both subjective and objective cognitive outcomes [22]. However, single-component interventions, particularly cognitive training, may show limited generalization to everyday functioning [19,23,24]. Similarly, psychological and mind–body interventions have demonstrated efficacy in alleviating emotional distress, fatigue, and improving quality of life, yet they typically target specific symptoms rather than the multidimensional profile of CRCI [27,32,33,35,36,37].

In this integrative survivorship program, we combine approaches from psychoeducation, cognitive behavioral therapy, neurocognitive strategy, function training and onco-yoga. By targeting both cognitive and psychological factors in an integrative therapy, we found a synergistic effect through a reciprocal reinforcement. This finding is consistent with improvements observed in exploratory variables such as perfectionism, procrastination, and symptoms of depression and anxiety (Table S8). It also aligns with previous research that shows strong associations between subjective cognitive complaints and psychological factors, while weak associations exist between objective neurocognitive impairment and psychological factors [19,20,21]. Since improvements were also observed in objective neurocognitive functioning, the cognitive gains cannot be solely attributed to reductions in anxio-depressive symptoms.

Moreover, a recent network meta-analysis supports the use of multimodal, therapist-guided interventions involving cognitive rehabilitation or strategy training and psychoeducation in group settings compared to single-component interventions to tackle CRCI, based on evidence from a single group-based program [54,55]. Our intervention extends this approach by additionally targeting psychological processes and functional goal transfer. By incorporating personalized goals and structured homework assignments, INCRT explicitly aims to promote transfer to daily functioning. Notably, most CRCI intervention studies focus on short-term neurocognitive outcomes, with limited evaluation of long-term effects or functional improvements in daily life [19,56]. The sustained improvements observed at 6-month follow-up in our study suggest the added value of an integrative approach that promotes generalization to everyday functioning as well as durability of effects.

Improvements in everyday activities (e.g., household tasks, reading, following conversations) were more frequent at the 6-month follow-up, suggesting a progressive internalization of learned strategies into real-life contexts. Interestingly, the survivorship program also shows potential in supporting work resumption, with 13 patients out of the 27 patients who were on sick leave at baseline being either in a return-to-work process or having increased their work capacity at post-intervention. Work resumption was the most frequently predefined personalized goal, and all patients who had this goal achieved it at the 6-month follow-up (Table S7). Cognitive complaints are reported as a key obstacle to return-to-work in cancer survivors [7], yet return-to-work outcomes are not routinely assessed in cognitive intervention studies.

Our findings regarding the improvement in cognitive, psychological and daily functioning can be explained by the emphasis on strategy use and individualized goals, which appear to strengthen individuals’ confidence in their cognitive abilities in daily life and, in turn, contribute to the sustained effects of the intervention on cognitive, psychological, and socio-professional functioning. This is consistent with the significant improvements found on the cognitive confidence subscale of the Metacognitions Questionnaire. Moreover, 12 patients also predefined self-care as an objective for the intervention. By achieving their self-care objectives, patients may have experienced a greater sense of control and well-being, which in turn could have reduced psychological distress and fatigue.

Furthermore, FCR, as an exploratory variable, did not show a significant reduction in severity (Table S8). The INCRT program includes only one psychoeducational session addressing FCR, suggesting that a more targeted intervention may be necessary to achieve improvements in this outcome.

Of interest is that the two glioma patients in our sample also improved on objective neurocognitive functioning, indicating the clinical need and utility to include CNS tumors in clinical interventions targeting cognition. Most often in research, CNS tumors are excluded from interventions due to reasons of heterogeneity. However, in this study, we chose to include patients with a history of CNS tumors to better align with real-world clinical needs.

Finally, the group-based format may not suit all survivors. Patients who were not mentally or physically fit to follow group sessions were referred to individual care. The two patients with a CNS tumor in our study reported that the INCRT program was overly fatiguing, suggesting that a less intensive, tailored group format for this population would be of clinical importance. This highlights the importance of offering personalized approaches that accommodate patients’ needs and capacities.

### 4.1. Strengths and Limitations

Strengths include a longitudinal design, comprehensive neuropsychological assessment alongside validated PROMs, and delivery of a multimodal intervention within routine clinical care, aligning with European recommendations from the Innovative Partnership for Action Against Cancer initiative for CRCI management [9]. Our primary goal was to test feasibility and effectiveness in a real-world, cross-diagnosis setting, which the results support.

Limitations include a modest sample and the absence of a control group. The design does not isolate specific components, as the study focused on the effects of an integrative and multidisciplinary approach.

### 4.2. Future Directions

Future research should incorporate neuroimaging to elucidate the neural mechanisms underlying intervention-related changes. Tailored INCRT programs for CNS tumor survivors may be clinically relevant and should be investigated, for example, by reducing session duration to better accommodate mental fatigue. Further development of individualized approaches for patients who are not suited to group-based formats is also warranted. Controlled studies are needed to strengthen the evidence base. Feasibility studies examining implementation across different hospitals and countries would enhance generalizability. Finally, as FCR did not improve following the INCRT program despite the inclusion of one psychoeducational session targeting FCR, these findings may indicate that a dedicated intervention specifically addressing FCR is required.

## 5. Conclusions

This multidisciplinary integrative survivorship program proved effective in enhancing both objective and subjective neurocognitive functioning, reducing emotional distress, fatigue, maladaptive metacognitions, and improving daily functioning and return-to-work among cancer survivors, with sustained benefits observed at the 6-month follow-up. We posit that these maintained improvements in cognitive, psychological, and socio-professional domains are supported by the integrative approach, which emphasizes metacognitive processes, personalized goals, strategy use, and their consistent application in daily life. These results underscore the feasibility and added value of a multidisciplinary integrative survivorship program that should be further implemented into routine clinical practice.

## Figures and Tables

**Table 1:** Biographic and medical characteristics at baseline.

	Median (Range) or *n* (%) (*N* = 38)
Age, years, median (range)	54 (30–77)
Gender, *n* (%)	
Female	27 (71)
Male	11 (29)
Education, *n* (%)	
Lower secondary school	2 (5)
Higher secondary school	12 (32)
Higher education	24 (63)
Civil status, *n* (%)	
Married or cohabiting	29 (76)
Single	8 (21)
Widowed	1 (3)
Work status, *n* (%)	
Part-time professionally active	9 (24)
Full-time professionally active	5 (13)
Part-time sick leave	10 (26)
Full-time sick or disability leave	17 (45)
Retired	4 (11)
Retired and part-time professionally active	1 (3)
Tumor type, *n* (%)	
Breast cancer	15 (40)
Melanoma	10 (26) *
Hematological cancer	4 (11)
Gynecological cancer	2 (5)
Head & neck cancer	2 (5)
Colon cancer	2 (5)
Bladder cancer	1 (3)
Glioblastoma	1 (3)
Low-grade glioma	1 (3)
Stage, *n* (%)	
I	8 (21)
II	6 (16)
III	8 (21)
IV	9 (24)
Other	1 (3)
Not applicable	4 (11)
Unknown	2 (5)
Years since diagnosis, median (range)	3.50 (0.28–16.14)
Years since remission, median (range)	2.45 (0.04–16.10)
Oncological treatment (with possible combinations), *n* (%)	
Chemotherapy	25 (66)
Immunotherapy	12 (32)
Hormonal therapy	12 (32)
Targeted therapy	5 (13)
Stem cell transplant (incl. chemo for transplant)	2 (5)
Surgery	27 (71)
Radiotherapy	25 (66)
Financial difficulties, *n* (%)	
No	22 (58)
I have to watch my expenses	16 (42)
Family problems, *n* (%)	15 (40)
Relational problems	4 (11)
Concerns about partner in relation to disease	3 (8)
Concerns about children in relation to disease	3 (8)
Other concerns not related to disease	4 (11)
Not specified	1 (3)
Sleep problems, *n* (%)	25 (66)
Psychotropic medication, *n* (%)	10 (26)
Antidepressant	9 (24)
Sedative	1 (3)
Physical activity, *n* (%)	
Never	4 (10)
Rarely	6 (15)
Regularly	15 (39)
Sometimes	10 (26)
Intensively	3 (8)

Note. * One patient was subsequently diagnosed with a stage T1N0M0 triple negative breast cancer.

**Table 2:** Results from the different linear mixed models (LMMs) estimating objective NCF (model 1), subjective NCF (model 2), HADS-total (model 3), FSS (model 4), and the MCQ-30 (model 5), respectively.

	Estimate	SE	*p*	95% CI
**Model 1: Objective NCF**				
Within group effects				
Intercept	0.937	0.408	0.028	0.108 to 1.766
Time: T0 (pre) = reference category				
T1 (post)	0.323	0.054	<0.001	0.215 to 0.431
T2 (6-month FU)	0.398	0.074	<0.001	0.251 to 0.545
Effect of age *	−0.019	0.008	0.020	−0.035 to −0.003
Effect of education level (higher education = reference category) *				
Lower secondary school	−0.301	0.370	0.422	−1.054 to 0.452
Higher secondary school	−0.401	0.172	0.026	−0.751 to −0.052
Effect of group (8-week INCRT = reference category)				
12-week INCRT	−0.049	0.183	0.791	−0.418 to 0.321
Time × group interaction (8-week INCRT and T0 (pre) = reference categories)				
T1 (post) × 12-week INCRT	0.033	0.319	0.751	−0.155 to 0.214
T2 (6-month FU) × 12-week INCRT	0.033	0.126	0.793	−0.218 to 0.284
**Model 2: Subjective NCF**				
Within group effects				
Intercept	51.600	2.522	<0.001	46.557 to 56.643
Time: T0 (pre) = reference category				
T1 (post)	−9.480	1.962	<0.001	−13.393 to −5.567
T2 (6-month FU)	−11.680	2.557	<0.001	−16.759 to −6.601
Effect of group (8-week INCRT = reference category)				
12-week INCRT	−9.292	4.312	0.035	−17.914 to −0.670
Time × group interaction (8-week INCRT and T0 (pre) = reference categories)				
T1 (post) × 12-week INCRT	3.249	3.355	0.336	−3.441 to 9.939
T2 (6-month FU) × 12-week INCRT	6.065	4.371	0.169	−2.618 to 14.748
**Model 3: HADS-total**				
Within group effects				
Intercept	17.560	1.164	<0.001	15.240 to 19.880
Time: T0 (pre) = reference category				
T1 (post)	−2.560	1.078	0.020	−4.710 to −0.410
T2 (6-month FU)	−4.520	1.352	0.001	−7.203 to −1.837
Effect of group (8-week INCRT = reference category)				
12-week INCRT	−4.714	1.991	0.020	−8.680 to −0.748
Time × group interaction (8-week INCRT and T0 (pre) = reference categories)				
T1 (post) × 12-week INCRT	0.791	1.844	0.669	−2.886 to 4.467
T2 (6-month FU) × 12-week INCRT	1.828	2.311	0.431	−2.759 to 6.415
**Model 4: FSS**				
Within group effects				
Intercept	47.080	2.513	<0.001	42.062 to 52.098
Time: T0 (pre) = reference category				
T1 (post)	−8.960	2.063	<0.001	−13.074 to −4.846
T2 (6-month FU)	−9.400	2.661	<0.001	−14.684 to −4.116
Effect of group (8-week INCRT = reference category)				
12-week INCRT	−12.311	4.296	0.006	−20.890 to −3.731
Time × group interaction (8-week INCRT and T0 (pre) = reference categories)				
T1 (post) × 12-week INCRT	9.575	3.528	0.008	2.542 to 16.609
T2 (6-month FU) × 12-week INCRT	8.246	4.549	0.073	−0.788 to 17.280
**Model 5: MCQ-30**				
Within group effects				
Intercept	68.160	2.689	<0.001	62.771 to 73.549
Time: T0 (pre) = reference category				
T1 (post)	−3.640	1.865	0.055	−7.358 to 0.078
T2 (6-month FU)	−6.560	2.473	0.010	−11.476 to −1.644
Effect of group (8-week INCRT = reference category)				
12-week INCRT	−6.391	4.597	0.170	−15.605 to 2.824
Time × group interaction (8-week INCRT and T0 (pre) = reference categories)				
T1 (post) × 12-week INCRT	−2.745	3.188	0.392	−9.102 to 3.613
T2 (6-month FU) × 12-week INCRT	−3.055	4.229	0.472	−11.461 to 5.350

Note. * Age and education level were added to the model as controlling variables and are, therefore, not interpreted in the text. Pre = pre-INCRT evaluation; Post = post-INCRT evaluation; FU = follow-up evaluation. NCF = neurocognitive functioning; HADS = Hospital Anxiety and Depression Scale; FSS = Fatigue Severity Scale; MCQ-30 = Metacognitions Questionnaire.

**Table 3:** Number of patients improved at the post-INCRT (T1) and 6-month follow-up (T2) assessment per item of daily functioning assessment.

Item	Improvement at T1 (Post-INCRT) *N* = 38	Improvement at T2 (6-Month Follow-Up) *N* = 38
Managing household		
Household cleaning	9	16
Preparing easy meals	5	5
Preparing a complex recipe	9	12
Doing groceries	10	10
Administration and planning		
Administration and financial aspects	9	10
Follow planning	8	10
Coping with unexpected situations	15	15
Professional		
Professionally active—work capacity	3	9
Work on task calmly	7	7
Finish tasks	4	5
Keeping up with e-mails	4	4
Plans to start (voluntary) work	5	6
Leisure and reading		
Following TV series	11	11
Reading easy book	10	12
Reading complex novel/non-fiction	16	19
Reading journal or magazine	6	9
Single tasking	17	18
Sports and leisure activities		
Does sports activity	8	11
Does leisure activity	7	14
Social interactions		
Follow group conversation	10	17
Social relations	3	6
Setting boundaries	16	16
Perceived improvement of INCRT		
Changes in diet	12	17
Understanding of emotions and cognitions	38	38

**Table 4:** Descriptive statistics of patient-reported outcome measures (PROMs).

	Pre-INCRT (T0)	Post-INCRT (T1)	6-Month Follow-Up (T2)
CFQ			
Mean, sd	48.42 (14.62)	40.05 (12.91)	38.82 (10.79)
Number of patients with elevated levels of subjective cognitive complaints (*n*, %)	25 (65.8%)	14 (36.8%)	13 (34.2%)
HADS			
HADS-A			
Mean, sd	9.53 (4.20)	8.21 (4.50)	7.37 (3.75)
Number of patients with elevated levels of anxiety	28 (73.7%)	19 (50.0%)	19 (50.0%)
HADS-D			
Mean, sd	6.42 (3.31)	5.45 (3.12)	4.68 (2.52)
Number of patients with elevated levels of depression	13 (34.2%)	10 (26.3%)	6 (15.8%)
HADS Total			
Mean, sd	15.95 (6.17)	13.66 (6.45)	12.05 (5.61)
Number of patients with psychological distress (any subscale ≥ 8)	31 (81.6%)	21 (55.3%)	19 (50.0%)
FSS			
Mean, sd	42.87 (12.53)	37.18 (13.28)	36.29 (13.31)
Number of patients with elevated levels of fatigue	30 (78.9%)	22 (57.9%)	20 (52.6%)
MCQ-30			
Mean, sd	65.97 (14.78)	61.39 (13.50)	58.37 (13.00)
Number of patients with elevated levels of unhelpful metacognitive beliefs (z ≥ 1.645)	12 (31.6%)	8 (21.1%)	6 (15.8%)

Note. CFQ = Cognitive Failures Questionnaire; HADS = Hospital Anxiety and Depression Scale; FSS = Fatigue Severity Scale: MCQ-30 = Metacognition Questionnaire-30. A higher score on the CFQ, HADS, FSS and MCQ-30 indicates more symptomatology.

## Data Availability

The datasets generated and analyzed during the study are available from the corresponding author upon reasonable request.

## Supplementary Materials

The following supporting information can be downloaded at: https://www.mdpi.com/article/10.3390/cancers18050785/s1, Figure S1: Mean and standard error mean of the FSS for the 8-week and 12-week group per timepoint; Figure S2: Boxplots and *p*-values of the linear mixed models of the exploratory endpoints; Table S1: Overview of the tests included in the composite score of objective neurocognitive functioning; Table S2: Mean and standard deviation of neuropsychological tests at each timepoint; Table S3: Results of the Reliable Change Index per subtest for pre to post and pre to follow-up assessment; Table S4: neuropsychological evaluation using the Reliable Change Index for sub-analysis of glioma patients; Table S5: Linear mixed model analyses examining moderation of change in objective neurocognitive functioning by years since remission, age, gender, and education level; Table S6: Pearson correlation at T0 between patient-reported outcome measures and objective neurocognitive functioning; Table S7: Assessment of goals at the post and 6-month follow-up INCRT; Table S8: Linear mixed model analysis of exploratory variables.
