# Supplementary material for: The Effectiveness of a Multidisciplinary Integrative Survivorship Program for Cancer-Related Cognitive Impairment: A Prospective Cohort Study
Source: Cancers (Basel). 2026 Feb 28;18(5):785. doi: 10.3390/cancers18050785 (PMC12984640; doi:10.3390/cancers18050785)
Supplement: Supplementary file 1 [file cancers-18-00785-s001.zip › cancers-4141702-supplementary.pdf]

cancers MDPI

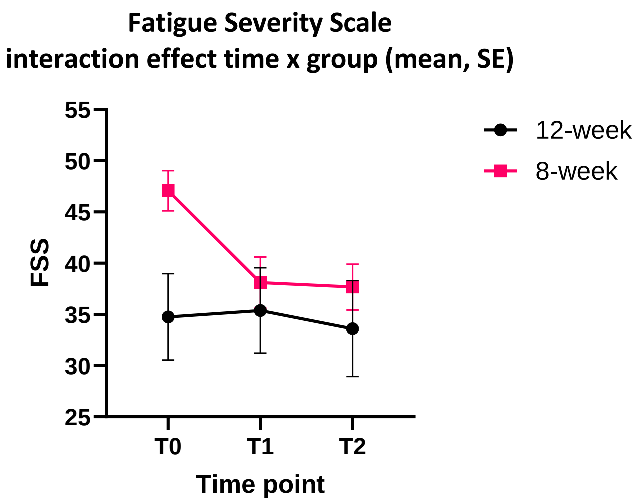

Figure S1. Mean and standard error mean of the FSS for the 8-week and 12-week group per timepoint.

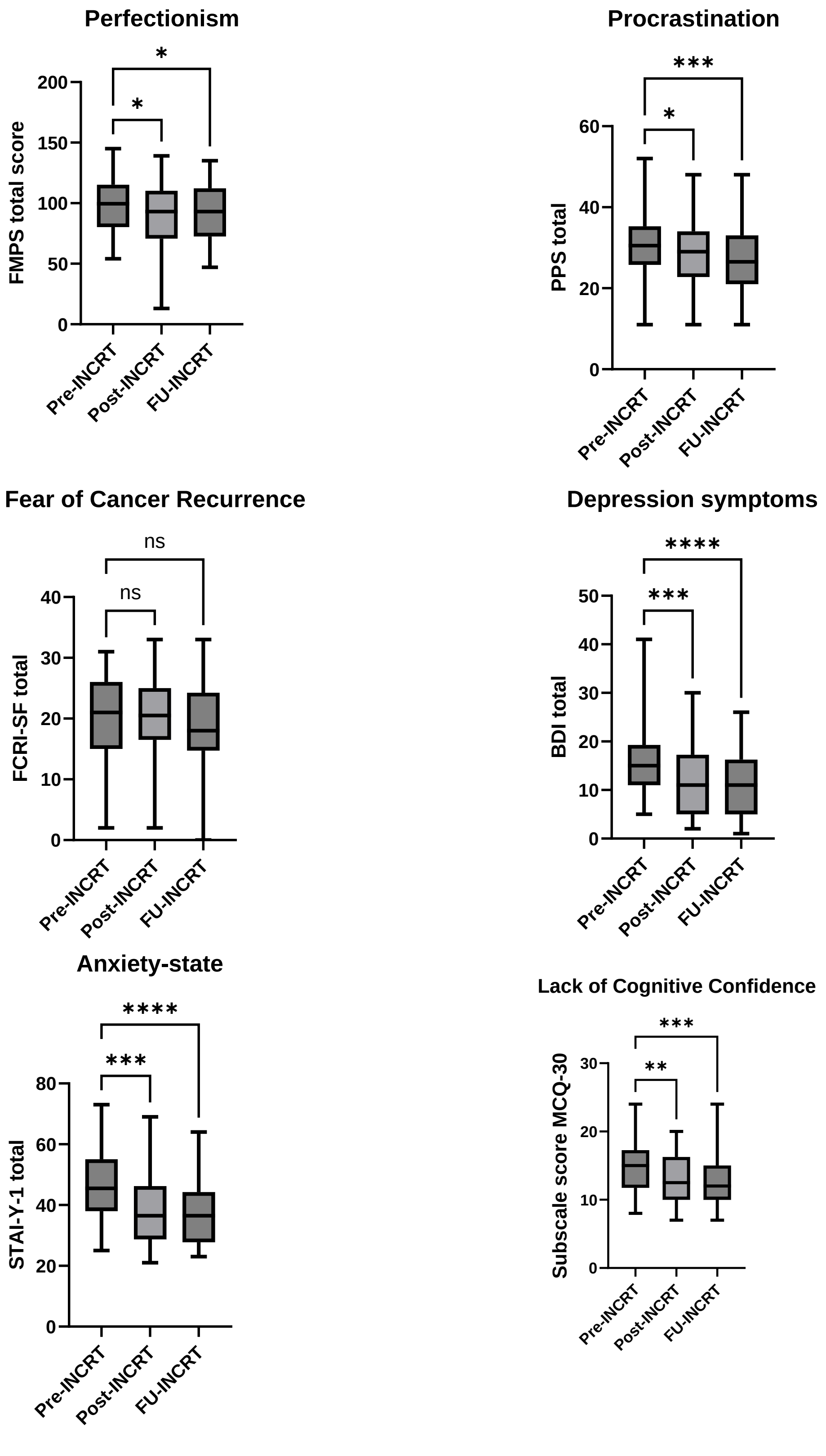

Figure S2. Boxplots and p-values of the linear mixed models of the exploratory endpoints.

Table S1. Overview of the tests included in the composite score of objective neurocognitive functioning.

Composite variable	Neuropsychological test (outcome measure)
Composite attention and processing speed	• Alertness (mean reaction time, log ms)<br>• Divided Attention (mean reaction time, log ms)<br>• Divided Attention – False Alarms (n)<br>• TMT-A Processing speed (s)<br>• WAIS-IV Symbol Search (n)<br>• WAIS-IV Coding (n)
Composite memory	• California Verbal Learning Test (CVLT-II) - Total Verbal Learning (n)<br>• California Verbal Learning Test (CVLT-II) - Verbal Short-Term Free Recall (n)<br>• California Verbal Learning Test (CVLT-II) - Verbal Long-Term Free Recall (n)<br>• Figural Memory Test - Total Visual Learning (n)<br>• Figural Memory Test - Visual Short-Term Recall (n)<br>• Figural Memory Test - Visual Long-Term recall (n)<br>• Figural Memory Test - Visual Long-Term Recognition (n)<br>• Corsi Block Tapping Test – Forwards span (n)<br>• WAIS-IV Digit Span Forward (n)
Composite executive functioning	• TMT-B Cognitive Flexibility (s)<br>• Tower of London Test - Planning Ability (n)<br>• N-Back Test – number correct (n)<br>• N-Back Test – number errors (n)<br>• Response Inhibition Test – commission errors (n)<br>• WAIS-IV Digit Span Backward (n)
Composite objective neurocognitive functioning	• Composite attention and processing speed<br>• Composite memory<br>• Composite executive functioning

Note. Internal consistency was calculated for all composite variables. We combined tests measuring attention and processing speed into one composite score because of an improved internal consistency when using the combination.

Table S2. Mean and standard deviation of neuropsychological tests at each timepoint.

Neuropsychological test	T0 Mean (SD)	T1 Mean (SD)	T2 Mean (SD)
Perception and attention functions test			
Alertness (mean reaction time, log ms)	252.39 (49.34)	241.63 (33.37)	236.19 (33.87)
Divided Attention (mean reaction time, log ms)	594.84 (161.21)	561.95 (109.52)	530.04 (103.69)
Divided Attention – False Alarms (n)	4.37 (5.19)	2.71 (2.71)	1.71 (1.64)
Trail Making Test			
TMT-A Processing speed (s)	23.46 (5.71)	22.20 (6.11)	21.50 (4.68)
TMT-B Cognitive Flexibility (s)	35.30 (12.06)	32.58 (11.54)	31.35 (9.88)

Figural Memory Test			
Total Visual Learning (n)	25.63 (8.91)	32.39 (8.36)	33.00 (8.24)
Visual Short-Term Recall (n)	6.63 (2.30)	7.76 (1.67)	7.61 (1.78)
Visual Long-Term Recall (n)	6.61 (2.40)	7.68 (1.54)	7.61 (1.93)
Visual Long-Term Recognition (n)	15.63 (2.28)	16.29 (1.97)	16.68 (1.93)
Tower of London Test			
Planning Ability (n)	15.32 (3.59)	15.76 (3.18)	15.34 (3.60)
N-Back Test			
Correct (n)	11.84 (2.56)	13.32 (2.06)	13.32 (2.20)
Errors (n)	5.32 (10.68)	3.71 (9.40)	2.92 (5.35)
Response Inhibition Test			
Inhibition	5.24 (3.48)	4.63 (3.13)	3.79 (2.32)
Corsi Block Tapping Test			
Corsi Block Forwards Span	4.79 (0.96)	4.95 (1.06)	4.97 (0.94)
California Verbal Learning Test II (CVLT-II)			
Total Verbal Learning (n)	53.87 (12.98)	59.61 (11.71)	58.97 (11.21)
Verbal Short-Term Free Recall (n)	11.42 (3.45)	12.87 (3.31)	13.05 (3.08)
Verbal Long-Term Free Recall (n)	11.76 (3.85)	13.11 (3.57)	12.97 (2.99)
WAIS-IV			
Digit Span Forward (n)	5.50 (1.01)	5.74 (1.20)	5.61 (0.97)
Digit Span Backward (n)	4.13 (0.99)	4.53 (1.08)	4.50 (0.95)
Symbol Search (n)	29.50 (6.64)	30.95 (6.71)	32.76 (7.34)
Coding (n)	63.66 (12.30)	67.16 (13.66)	67.95 (14.85)

Note. The following tests are part of the computerized neurocognitive test battery COGBAT® and CORSI®: Perception and attention functions test, Trail Making Test, Figural Memory Test, Tower of London Test, N-Back Test, Response Inhibition Test, Corsi Block Tapping Test; SD = Standard Deviation; log ms = logarithmic milliseconds; n = count.

Table S3. Results of the Reliable Change Index per subtest from pre to post and pre to follow-up assessment.

Neuropsychological Test	Comparison	Deterioration	Stable	Improvement
Processing Speed				
TMT-A	Pre-Post	1 (2.6%)	29 (76.3%)	8 (21.1%)
	Pre-FU	2 (5.3%)	29 (76.3%)	7 (18.4%)
WAIS-IV Symbol Search	Pre-Post	0 (0.0%)	37 (97.4%)	1 (2.6%)
	Pre-FU	0 (0.0%)	37 (97.4%)	1 (2.6%)
WAIS-IV Coding	Pre-Post	0 (0.0%)	37 (97.4%)	1 (2.6%)
	Pre-FU	0 (0.0%)	36 (94.7%)	2 (5.3%)
Attention				
Perception and attention functions test - Alertness	Pre-Post	6 (15.8%)	25 (65.8%)	7 (18.4%)
	Pre-FU	4 (10.5%)	25 (65.8%)	9 (23.7%)

Perception and attention functions test - Divided Attention	Pre-Post<br>Pre-FU	2 (5.3%)<br>0 (0.0%)	31 (81.6%)<br>31 (81.6%)	5 (13.2%)<br>7 (18.4%)
Memory				
FMT - Learning Ability	Pre-Post	1 (2.6%)	21 (55.3%)	16 (42.1%)
	Pre-FU	0 (0.0%)	19 (50.0%)	19 (50.0%)
FMT - Short-term memory	Pre-Post	0 (0.0%)	35 (92.1%)	3 (7.9%)
	Pre-FU	1 (2.6%)	31 (81.6%)	6 (15.8%)
FMT - Long-term memory	Pre-Post	0 (0.0%)	35 (92.1%)	3 (7.9%)
	Pre-FU	1 (2.6%)	33 (86.8%)	4 (10.5%)
FMT - Recognition	Pre-Post	1 (2.6%)	33 (86.8%)	4 (10.5%)
	Pre-FU	0 (0.0%)	34 (89.5%)	4 (10.5%)
CVLT-II– Learning ability	Pre-Post	0 (0.0%)	31 (81.6%)	7 (18.4%)
	Pre-FU	0 (0.0%)	35 (92.1%)	3 (7.9%)
CVLT-II – Short-term free recall	Pre-Post<br>Pre-FU	1 (2.6%)<br>0 (0.0%)	33 (86.8%)<br>33 (86.8%)	4 (10.5%)<br>5 (13.2%)
CVLT-II – Long-term delayed free recall	Pre-Post<br>Pre-FU	0 (0.0%)<br>0 (0.0%)	33 (86.8%)<br>35 (92.1%)	5 (13.2%)<br>3 (7.9%)
WAIS-IV Digit Span Forward	Pre-Post	7 (18.4%)	21 (55.3%)	10 (26.3%)
	Pre-FU	8 (21.1%)	20 (52.6%)	10 (26.3%)

Table S3 (continued). Results of the Reliable Change Index per subtest for pre to post and pre to follow-up assessment.

Neuropsychological Test	Comparison	Deterioration	Stable	Improvement
Corsi Block Tapping Test	Pre-Post	1 (2.6%)	33 (86.8%)	4 (10.5%)
	Pre-FU	1 (2.6%)	36 (94.7%)	1 (2.6%)
Executive functioning				
TMT-B	Pre-Post	2 (5.3%)	34 (89.5%)	2 (5.3%)
	Pre-FU	2 (5.3%)	33 (86.8%)	3 (7.9%)
Response Inhibition	Pre-Post	3 (7.9%)	32 (84.2%)	3 (7.9%)
	Pre-FU	2 (5.3%)	29 (76.3%)	7 (18.4%)
N-Back Test	Pre-Post	0 (0.0%)	31 (81.6%)	7 (18.4%)
	Pre-FU	1 (2.6%)	32 (84.2%)	5 (13.2%)
WAIS-IV Digit Span Backward	Pre-Post<br>Pre-FU	7 (18.4%)<br>4 (10.5%)	17 (44.7%)<br>21 (55.3%)	14 (36.8%)<br>13 (34.2%)
Planning Ability	Pre-Post<br>Pre-FU	2 (5.3%)<br>3 (7.9%)	31 (81.6%)<br>31 (81.6%)	5 (13.2%)<br>4 (10.5%)

Note. FMT = Figural Memory Test; TMT = Trail Making Test; CVLT-II = California Verbal Learning Test.

Table S4. Neuropsychological evaluation using the Reliable Change Index for sub-analysis of glioma patients.

**Deficit at T0 (z≤-1.5)**	**Reliable Change at T1 compared to T0**	**Reliable Change at T2 compared to T0**
**Case 1. High-grade glioma**		
Processing speed TMT-A	No reliable change	No reliable change
FGT visual learning ability	**Reliable improvement**	No reliable change
FGT visual short-term recall	No reliable change	**Reliable improvement**
FGT visual long-term recall	No reliable change	**Reliable improvement**
CVLT verbal learning ability	No reliable change	No reliable change
CVLT short-term recall	No reliable change	No reliable change

CVLT long-term recall	No reliable change	**Reliable improvement**
Cognitive flexibility TMT-B	No reliable change	No reliable change
Working memory N-back test	**Reliable improvement**	No reliable change
**Deficit at T0 (z≤-1.5)**	**Reliable Change at T1 compared to T0**	**Reliable Change at T2 compared to T0**
**Case 2. Low-grade glioma**		
FGT visual learning ability	No reliable change	**Reliable improvement**
FGT visual long-term recall	No reliable change	No reliable change
FGT visual long-term recognition	No reliable change	**Reliable improvement**
CVLT long-term recall	**Reliable improvement**	No reliable change

Note. The table depicts the Reliable Change Index for the neuropsychological tests for which the patients had a deficit at T0. Case 1 (high-grade glioma): Case 1 had neurocognitive impairment according to ICCTF guidelines at all timepoints. The Cognitive Failures Questionnaire (CFQ) showed cognitive complaints below the clinical cutoff (≥ 44) at all timepoints (CFQ total score at T0 = 43; T1 = 39; T2 = 34). Case 2 (low-grade glioma): Case 2 had neurocognitive impairment according to ICCTF guidelines at T0 and T1, but not at T2. The Cognitive Failures Questionnaire (CFQ) showed cognitive complaints above the clinical cutoff at all timepoints (CFQ total score at T0 = 54; T1 = 56; T2 = 47).

Table S5. Linear mixed model analyses examining moderation of change in objective neurocognitive functioning by years since remission, age, gender, and education level.

**A. Fixed effects estimates examining change in objective neurocognitive functioning and moderation by years since remission**				
Predictor	β	SE	*p*	95% CI
Intercept	0.988	0.411	.022	0.151 to 1.824
Time: T0 (pre) = reference category				
T1 (post)	0.232	0.058	<.001	0.116 to 0.348
T2 (6-month FU)	0.342	0.080	<.001	0.184 to 0.500
Effect of education level (higher education = reference category)				
Lower secondary school	-0.335	0.356	.354	-1.058 to 0.389
Higher secondary school	-0.383	0.178	.039	-0.746 to -0.021
Effect of age	-0.019	0.008	.019	-0.035 to -0.003
Effect of years since remission	-0.022	0.027	.404	-0.076 to 0.031
**Moderation analysis of time × years since remission (T0 = reference category)**				
**T1 (post) × years since remission**	0.033	0.013	**.014**	0.007 to 0.059
T2 (6-month FU) × years since remission	0.022	0.018	.223	-0.014 to 0.058

**B. Fixed effects estimates examining change in objective neurocognitive functioning and moderation by age**				
Predictor	β	SE	*p*	95% CI
Intercept	0.947	0.430	.033	0.080 to 1.813
Time: T0 (pre) = reference category				
T1 (post)	0.377	0.231	.107	-0.084 to 0.837
T2 (6-month FU)	0.373	0.314	.238	-0.252 to 0.999
Effect of education level (higher education = reference category)				
Lower secondary school	-0.321	0.348	.363	-1.028 to 0.387
Higher secondary school	-0.402	0.169	.024	-0.746 to -0.057
Effect of age	-0.020	0.008	.020	-0.036 to -0.003
**Moderation analysis of time × age (T0 = reference category)**				
T1 (Post) × Age	-0.001	0.004	.848	-0.009 to 0.008

T2 (6-month FU) × Age	0.001	0.006	.907	-0.011 to 0.012
**C. Fixed effects estimates examining change in objective neurocognitive functioning and moderation by gender**				
Predictor	β	SE	*p*	95% CI
Intercept	0.922	0.448	1.00	-3.263 to 5.106
Time: T0 (pre) = reference category				
T1 (Post)	0.400	0.049	<.001	0.301 to 0.498
T2 (6-month FU)	0.438	0.067	<.001	0.303 to 0.572
Effect of education level (higher education = reference category)				
Lower secondary school	-0.329	0.358	.365	-1.057 to 0.399
Higher secondary school	-0.407	0.175	.027	-0.764 to -0.050
Effect of age	-0.020	0.008	.018	-0.036 to -0.004
Effect of gender (female = reference category)				
Male	0.093	0.188	.622	-0.286 to 0.472
**Moderation analysis of time × gender (T0 and female = reference category)**				
**T1 (Post) × Male**	-0.230	0.092	**.014**	-0.413 to -0.047
T2 (6-month FU) × Male	-0.097	0.125	.443	-0.346 to 0.153
**D. Fixed effects estimates examining change in objective neurocognitive functioning and moderation by education level**				
Predictor	β	SE	*p*	95% CI
Intercept	0.967	0.401	.021	0.152 to 1.781
Time: T0 (pre) = reference category				
T1 (post)	0.293	0.055	<.001	0.182 to 0.403
T2 (6-month FU)	0.343	0.075	<.001	0.193 to 0.492
Effect of education level (higher education = reference category)				
Lower secondary school	-0.423	0.373	.263	-1.175 to 0.329
Higher secondary school	-0.492	0.181	.009	-0.858 to -0.127
Effect of age	-0.019	0.008	.015	-0.035 to -0.004
**Moderation analysis of time × education level (T0 and higher education = reference category)**				
T1 (Post) × Lower secondary school	0.148	0.199	.459	-0.249 to 0.546
T2 (6-month FU) × Lower secondary school	0.197	0.271	.469	-0.342 to 0.737
T1 (Post) × Higher secondary school	0.104	0.096	.283	-0.087 to 0.295
T2 (6-month FU) × Higher secondary school	0.180	0.130	.172	-0.080 to 0.439

Note. Age and education were included as covariates in the primary model. The present analyses examined whether years since remission (Model A), age (Model B), gender (Model C), and education level (Model D) moderated change over time. Four separate linear mixed models were conducted. Each model included fixed effects for time, age, and education (highest level as reference), and the respective time × moderator interaction term (i.e., time × years since remission, time × age, time × gender, or time × education level). FU = follow-up.

Table S6. Pearson correlation at T0 between patient-reported outcome measures and objective neurocognitive functioning.

**Variable**	**Composite NCF**	**MCQ**	**CFQ**	**FSS**	**HADS-A**	**HADS-D**
Composite NCF	—					
MCQ	.10	—				
CFQ	.33*	.35*	—			
FSS	.35*	.39*	.58**	—		
HADS-A	.10	.60**	.44**	.46**	—	
HADS-D	.13	.19	.34*	.48**	.34*	—

Note. A higher score on the MCQ, CFQ, FSS, HADS-A, and HADS-D corresponds to a higher level of symptomatology. A higher score on the composite NCF corresponds to a better performance on the neuropsychological tests. * $p < .05$. ** $p < .01$. *** $p < .001$.

Table S7. Assessment of goals at post and 6-month follow-up INCRT.

**Numbers of patients (%) who achieved at least 1 of the goals**						**Post: 38 (100%)<br>Follow-up (FU): 38 (100%)**			
Category	Work resumption	Functioning at (voluntary)work	Administration	Hobby	Sport activity	Household work	Self-care	Expanding social contacts	Communication
Numbers of patients who had the goal (% out of the full sample)	14 (36.8% out of total sample; 51.9% out of the 27 patients who were in the ability to increase work capacity)	12 (31.6%)	4 (10.5%)	14 (36.8%)	12 (31.6%)	9 (23.7%)	12 (31.6%)	5 (13.2%)	3 (7.9%)
Numbers of patients who achieved the goal (% among patients who had the goal)	Post: 12 (85.6%)<br>- In process: 10<br>- Increased work percentage: 2<br>- Work resumption: 0[a]<br><br>FU: 14 (100%)<br>- In process: 6<br>- Increased work percentage: 4<br>- Work resumption: 4[a]	Post: 12 (100%)[b]<br><br>FU: 12 (100%)[b]	Post: 3 (75%)<br><br>FU: 2 (50%)	Post: 12 (85.6%)<br><br>FU: 13 (92.9%)	Post: 12 (100%)<br><br>FU: 12 (100%)	Post: 9 (100%)<br><br>FU: 9 (100%)	Post: 10 (83.3%)<br><br>FU: 11 (91.7%)	Post: 5 (100%)<br><br>FU: 5 (100%)	Post: 2 (66.7%)[c]<br><br>FU: 2 (66.7%)[c]

Note. Patients could have more than one goal. Work resumption achievement was defined as any steps taken towards work resumption (such as having consultation with social nurses, job coaches, having job interviews, or work resumption itself). "In process" was defined as taking any steps towards job resumption but not having resumed work yet. [a.] One patient planned full-time work resumption before starting the INCRT program. This resulted in a 100% work resumption at post and follow-up without it being a specific goal during the program. Instead, the goal was to improve functioning at work. This patient was not included in the number of patients with 'return to work' as a goal. [b.] One patient had a goal of improving functioning at her voluntary work. All others were aimed at functioning at work in a professional context. [c.] One patient did not achieve the goal at post but did achieve the goal at follow-up, another patient achieved the goal at post but could not maintain this goal at follow-up.

Table S8. Linear mixed model analysis of exploratory variables.

	Type III Tests of Fixed Effects		Estimated marginal means			
	F	*p*	mean	SE	95% CI	Interpretation
BDI (depression symptoms)						
Time						
T0	10.838	<.001	15.689	1.214	13.265-18.113	Significant improvement over time
T1			11.066	1.214	8.642-13.490	
T2			10.874	1.214	8.450-13.298	
FMPS (perfectionism)						
Time						
T0	5.312	.007	99.725	4.144	91.411-108.788	Significant improvement over time
T1			89.323	4.144	81.039-97.607	
T2			92.505	4.144	84.221-100.788	
PPS (procrastination)						
Time						
T0	9.535	<.001	31.111	1.478	28.147-34.074	Significant improvement over time
T1			27.408	1.478	24.444-30.371	
T2			25.697	1.478	22.733-28.661	
STAI-Y-1 (anxiety-state)						
Time						
T0	11.249	<.001	44.086	1.838	40.421-47.752	Significant improvement over time
T1			36.694	1.838	33.028-40.359	
T2			36.163	1.838	32.497-39.829	
FCRI-SF (fear of cancer recurrence)						
Time						
T0	1.224	.300	19.214	1.208	16.787-21.641	No significant change over time
T1			19.423	1.208	16.996-21.850	
T2			18.308	1.208	15.881-20.734	
Lack of Cognitive Confidence subscale of MCQ-30						
Time						
T0	7.513	.001	14.921	.630	13.665-16.178	Significant change over time
T1			13.026	.630	11.770-14.283	
T2			12.605	.630	11.349-13.862	

Note. Results from linear mixed models estimating the intervention effects on the exploratory variables of depression symptoms (BDI), perfectionism (FMPS), procrastination (PPS), anxiety-state (STAI-Y-1), and Fear of Cancer Recurrence (FCRI-SF), and lack of cognitive confidence subscale of the metacognitions questionnaire (MCQ-30).
